# Screening for atrial fibrillation using an ECG monitoring patch among the elderly population in rural China: a single-arm study

**DOI:** 10.1093/europace/euag135

**Published:** 2026-06-08

**Authors:** Le Zhou, Yize Zhao, Xingxing He, Jue Wang, Fadong Li, Xiangyi Kong, Yinggang Wu, Mengge Zhou, Shaobin Wei, Liu He, Chao Jiang, Xin Li, Shijun Xia, Xiaoxia Liu, Xueyuan Guo, Ning Zhou, Songnan Li, Cheng Yu, Chenxi Jiang, Ribo Tang, Song Zuo, Caihua Sang, Deyong Long, Xin Du, Jianzeng Dong, Changsheng Ma

**Affiliations:** Department of Cardiology, Beijing Anzhen Hospital, Capital Medical University, National Clinical Research Center for Cardiovascular Diseases, 2 Anzhen Road, Chaoyang District, Beijing 100029, People’s Republic of China; Department of Cardiology, Beijing Anzhen Hospital, Capital Medical University, National Clinical Research Center for Cardiovascular Diseases, 2 Anzhen Road, Chaoyang District, Beijing 100029, People’s Republic of China; Department of Cardiology, Second People's Hospital of Quzhou, Zhejiang 324000, China; Department of Cardiology, Beijing Anzhen Hospital, Capital Medical University, National Clinical Research Center for Cardiovascular Diseases, 2 Anzhen Road, Chaoyang District, Beijing 100029, People’s Republic of China; Department of Cardiology, Beijing Anzhen Hospital, Capital Medical University, National Clinical Research Center for Cardiovascular Diseases, 2 Anzhen Road, Chaoyang District, Beijing 100029, People’s Republic of China; Department of Cardiology, Beijing Anzhen Hospital, Capital Medical University, National Clinical Research Center for Cardiovascular Diseases, 2 Anzhen Road, Chaoyang District, Beijing 100029, People’s Republic of China; Department of Cardiology, Second People's Hospital of Quzhou, Zhejiang 324000, China; Department of Epidemiology and Biostatistics, School of Basic Medicine, Peking Union Medical College, Chinese Academy of Medical Sciences, Beijing 100005, China; Department of Cardiology, Beijing Anzhen Hospital, Capital Medical University, National Clinical Research Center for Cardiovascular Diseases, 2 Anzhen Road, Chaoyang District, Beijing 100029, People’s Republic of China; Department of Cardiology, Beijing Anzhen Hospital, Capital Medical University, National Clinical Research Center for Cardiovascular Diseases, 2 Anzhen Road, Chaoyang District, Beijing 100029, People’s Republic of China; Department of Cardiology, Beijing Anzhen Hospital, Capital Medical University, National Clinical Research Center for Cardiovascular Diseases, 2 Anzhen Road, Chaoyang District, Beijing 100029, People’s Republic of China; Department of Cardiology, Beijing Anzhen Hospital, Capital Medical University, National Clinical Research Center for Cardiovascular Diseases, 2 Anzhen Road, Chaoyang District, Beijing 100029, People’s Republic of China; Department of Cardiology, Beijing Anzhen Hospital, Capital Medical University, National Clinical Research Center for Cardiovascular Diseases, 2 Anzhen Road, Chaoyang District, Beijing 100029, People’s Republic of China; Department of Cardiology, Beijing Anzhen Hospital, Capital Medical University, National Clinical Research Center for Cardiovascular Diseases, 2 Anzhen Road, Chaoyang District, Beijing 100029, People’s Republic of China; Department of Cardiology, Beijing Anzhen Hospital, Capital Medical University, National Clinical Research Center for Cardiovascular Diseases, 2 Anzhen Road, Chaoyang District, Beijing 100029, People’s Republic of China; Department of Cardiology, Beijing Anzhen Hospital, Capital Medical University, National Clinical Research Center for Cardiovascular Diseases, 2 Anzhen Road, Chaoyang District, Beijing 100029, People’s Republic of China; Department of Cardiology, Beijing Anzhen Hospital, Capital Medical University, National Clinical Research Center for Cardiovascular Diseases, 2 Anzhen Road, Chaoyang District, Beijing 100029, People’s Republic of China; Department of Neurosurgery, Second People's Hospital of Quzhou, 338 Xin’an Avenue, Qujiang District, Quzhou, Zhejiang 324000, People’s Republic of China; Department of Cardiology, Beijing Anzhen Hospital, Capital Medical University, National Clinical Research Center for Cardiovascular Diseases, 2 Anzhen Road, Chaoyang District, Beijing 100029, People’s Republic of China; Department of Cardiology, Beijing Anzhen Hospital, Capital Medical University, National Clinical Research Center for Cardiovascular Diseases, 2 Anzhen Road, Chaoyang District, Beijing 100029, People’s Republic of China; Department of Cardiology, Beijing Anzhen Hospital, Capital Medical University, National Clinical Research Center for Cardiovascular Diseases, 2 Anzhen Road, Chaoyang District, Beijing 100029, People’s Republic of China; Department of Cardiology, Beijing Anzhen Hospital, Capital Medical University, National Clinical Research Center for Cardiovascular Diseases, 2 Anzhen Road, Chaoyang District, Beijing 100029, People’s Republic of China; Department of Cardiology, Beijing Anzhen Hospital, Capital Medical University, National Clinical Research Center for Cardiovascular Diseases, 2 Anzhen Road, Chaoyang District, Beijing 100029, People’s Republic of China; Department of Cardiology, Beijing Anzhen Hospital, Capital Medical University, National Clinical Research Center for Cardiovascular Diseases, 2 Anzhen Road, Chaoyang District, Beijing 100029, People’s Republic of China; Department of Cardiology, Beijing Anzhen Hospital, Capital Medical University, National Clinical Research Center for Cardiovascular Diseases, 2 Anzhen Road, Chaoyang District, Beijing 100029, People’s Republic of China; Department of Cardiology, Beijing Anzhen Hospital, Capital Medical University, National Clinical Research Center for Cardiovascular Diseases, 2 Anzhen Road, Chaoyang District, Beijing 100029, People’s Republic of China

## Introduction

The prevalence of atrial fibrillation (AF) rises with age, yet its paroxysmal and asymptomatic nature leads to significant underdiagnosis.^1^ Conventional screening methods often fail to improve the detection rate or clinical outcome.^2^ Although AF screening has been widely investigated, evidence from large-scale screening studies in elderly populations from rural China, especially among low- and middle-income groups with limited healthcare access, remains scarce.^3^ In this study, we evaluated the performance of a seven-day single-lead electrocardiogram (ECG) patch against conventional 12-lead ECG in a large rural elderly cohort, with the aim of providing real-world evidence for AF screening in an underrepresented population.

## Methods

This prospective, single-arm study (NCT07149688) establishes the methodological framework for the subsequent GEMINI trial (NCT06842147). The study protocol was reviewed and approved by the Ethics Committee of the Second Hospital of Quzhou (Approval No. 2025 −71), and written informed consent was obtained from all participants before enrollment. Potential participants were initially identified via local community health records in Quzhou, China, with eligibility (age ≥ 60 years; no prior AF history) subsequently verified by local health workers during in-person medical history reviews. Following a baseline 12-lead ECG, all participants received a single-use ECG patch (Chengdu Xinjikang Technology Co., Ltd., Chengdu, China) for up to seven days of continuous monitoring. AF detection utilized a proprietary deep-learning algorithm designed to identify irregular RR intervals and absent P-waves. All AI-identified cases were subsequently reviewed and adjudicated by board-certified cardiologists. Participants with newly detected AF were referred within the local medical consortium for further evaluation and treatment, with monthly follow-up by village doctors to support medication adherence.

## Results

**Figure 1:**
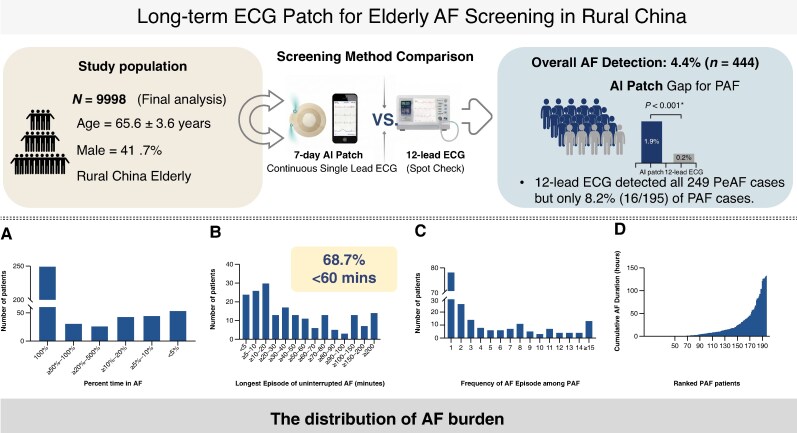
Screening for atrial fibrillation using an ECG monitoring patch among the elderly population in rural China: a single-arm study. Comparison of screening methods and AF burden distribution. The upper panel illustrates the study design involving 9998 elderly participants in rural China (mean age 65.6 ± 3.6 years). It compares a 7-day continuous single-lead ECG patch against a standard 12-lead ECG (spot check). While both methods identified all persistent AF (PeAF) cases, the AI patch significantly outperformed the 12-lead ECG in detecting paroxysmal AF (PAF), capturing 1.9% vs. 0.2% respectively (*P* < 0.001).The lower panel (*A–D*) details the AF burden distribution among detected patients:(A) Percentage of time spent in AF; (*B*) Duration of the longest uninterrupted AF episode; (*C*) Frequency of AF episodes among PAF patients; (*D*) Cumulative AF duration across ranked PAF patients. Abbreviations: AF = atrial fibrillation; ECG = electrocardiogram; PAF = paroxysmal atrial fibrillation; PeAF = persistent atrial fibrillation; *n* = Number of subjects.

Of 10 033 enrolled participants, 9998 provided analyzable data (mean age 65.6 ± 3.6 years; 41.7% male), as detailed in *Figure 1*. The median monitoring duration was 4.3 days (interquartile range [IQR]: 3.5–5.4), and the wear time effectiveness (defined as analyzable ECG duration/total monitoring duration×100%) was 97.2%. Newly diagnosed AF was detected in 444 participants (4.4%; mean age 76.4 ± 8.0 years; 52.5% male) via continuous patch monitoring, with a median CHA_2_DS_2_-VASc score of 2 (IQR: 2–3). Patch monitoring detected significantly more cases than the baseline 12-lead ECG (4.4% vs. 2.7%; *P* < 0.001). All 249 cases of persistent AF (PeAF) were captured by both methods. However, the baseline 12-lead ECG captured only 16 cases (0.2%) of paroxysmal atrial fibrillation (PAF); conversely, continuous monitoring identified 195 (1.9%), representing a tenfold increase in detection rate. The median AF burden (defined as the ratio of AF duration to analyzable ECG duration) in AF participants was 96.8% (IQR: 13.2–99.8%). However, for participants with PAF, the median AF burden was 10.0% (IQR: 4.6–24.8%), as shown in *Figure 1A*.

In the analysis of the PAF subgroup, several key patterns regarding arrhythmia burden and clinical correlation emerged. Notably, 68.7% of these patients had a longest uninterrupted episode duration of less than 60 min (*Figure* 1*B*). The frequency of episodes varied: 40.0% (*n* = 78) of participants experienced only a single episode, 13.3% (*n* = 26) had two, and nearly half (46.7%, *n* = 91) recorded three or more during the monitoring period (*Figure 1C*). Total AF duration for these individuals is further detailed in *Figure 1D*. Furthermore, AF burden showed a moderate positive correlation with the duration of the longest episode (Spearman's correlation coefficient = 0.35, *P* < 0.001). Age-stratified data revealed that the AF detection rate peaked at 15.9% in participants aged ≥90 years. In the multivariable model adjusted for all covariates, age ≥75 years, male sex, peripheral vascular disease, hyperlipidaemia, and congestive heart failure emerged as significant independent predictors for screen-detected AF.

## Discussion

To the best of our knowledge, this study represents the largest single-arm AF screening initiative in rural China to date, indicating that continuous AI-enabled ECG patch monitoring offers a superior diagnostic yield compared with conventional 12-lead ECG (4.4% vs. 2.7%). Particularly regarding patients with PAF, the patch monitoring achieved a tenfold higher diagnostic yield compared with standard ECG (1.9% vs. 0.2%).

The relatively high proportion of persistent AF and the substantial AF burden observed in our cohort may partly reflect limited prior exposure to healthcare and reduced opportunities for earlier AF detection in this rural elderly population, compared with cohorts enrolled in previous AF screening studies. Furthermore, the relatively high proportion of women in this cohort may partly reflect rural demographic patterns in this region, where many working-aged men migrate to urban areas for employment.

While conventional systematic and opportunistic screening methods often yield modest results or face implementation barriers, as seen in the D2AF and VITAL-AF trials, our approach leverages advanced temporal resolution to capture transient episodes.^4,5^ Notably, in our study, 68.7% of identified PAF episodes lasted less than one hour, highlighting a limitation of intermittent strategies.

Furthermore, this study also quantified AF burden, a metric increasingly recognized for its association with clinical outcomes such as ischaemic stroke.^6^ Unlike the GUARD-AF or STROKESTOP trials, which were conducted in higher-resource settings, our study provides real-world evidence on AF screening in an elderly population from rural China.^7–9^

## Conclusion

A 7-day single-lead ECG patch significantly enhanced AF detection among the rural elderly population, particularly for paroxysmal cases frequently missed by a one-off 12-lead ECG. With a 4.4% detection rate and a predominance of short-duration episodes, these findings highlight the limitations of intermittent screening. This scalable technology offers a robust strategy for early identification and risk stratification in resource-limited settings, warranting further research into its impact on cardiovascular morbidity and mortality.

## Data Availability

The data underlying this article will be shared on reasonable request to the corresponding author.
